# How Scientific Is Nursing? Answers From A New Characterization of Science

**DOI:** 10.1111/nup.70068

**Published:** 2026-02-23

**Authors:** Claus Beisbart, Maya Zumstein‐Shaha, Paul Hoyningen‐Huene

**Affiliations:** ^1^ Institute of Philosophy University of Bern Bern Switzerland; ^2^ School of Health Professions, Fachbereich Pflege Berner Fachhochschule Bern Switzerland; ^3^ Institute of Philosophy Leibniz University Hannover Hannover Germany

**Keywords:** classifications, consensus statement on emerging nursing knowledge, demarcation problem, nursing theories, scientific knowledge, systematicity

## Abstract

In the last few decades, nursing scholars have drawn on philosophy to establish the scientific status of nursing. However, well‐known philosophical accounts of science, such as those by Popper and Kuhn, are primarily targeted at the pure natural sciences. Accordingly, the application of such accounts to nursing has led to dubious results. In this paper, we propose a fresh start and apply Hoyningen‐Huene's recent account of science to nursing. According to Hoyningen‐Huene, knowledge about a given topic such as nursing is scientific if, and only if, it is more systematic than other knowledge about this topic. Here, systematicity manifests in various dimensions such as description, explanation, and defense of knowledge claims. In our application of Hoyningen‐Huene's account of science to nursing, we focus on current nursing practice and compare it with the state of nursing before the so‐called Consensus Statement on Emerging Nursing Knowledge. Upon examining the dimensions considered by Hoyningen‐Huene, we find that the knowledge currently used and gained by nurses is more systematic than it was before the Consensus Statement. For instance, in the dimension of prediction, knowledge has become more systematic through the use of classification systems. In the dimension of defending knowledge claims, systematicity has increased due to the use of measurement devices. According to our analysis, the scientific status of current nursing knowledge is partly due to the fact that nurses draw on knowledge from nursing science, a discipline that is organized like other scientific disciplines. Still, it is also the case that knowledge‐gaining practices in nursing are more systematic than they were before the Consensus Statement.

## 1 Introduction

Since the middle of the 19th century, there has been a strong quest to base nursing on the most recent available scientific knowledge and to ensure suitable education and training for nurses (e.g., Egenes 2017). For nursing to respond to the increasing challenges in healthcare, it was transformed from a craft into something like a science taught in academic institutions. Today, in most countries, prospective nurses can, or have to, study at a university or a university of applied sciences. In the context of this transformation, scholars working on nursing have stressed the scientific nature of nursing (e.g., Smith 2019). There is indeed no doubt that the scientific basis of nursing is essential for providing high‐quality care to meet the respective needs of patients.

To emphasize the scientific nature of nursing, scholars working on nursing have turned to the philosophy of science, an area of philosophy in which science, its products, and methods are systematically investigated. They have employed philosophical characterizations or theories of science and applied them to nursing, or at least to nursing science (e.g., Garrett 2018). While some authors have argued in an affirmative vein that nursing, or at least nursing science, is scientific according to this or that philosophical approach, others have used such an approach to criticize certain parts of nursing or nursing science as not yet scientific, or to make recommendations for future nursing.

Although the application of philosophy of science to nursing has led to interesting insights, related work has been fraught with problems. Two philosophers have frequently been consulted, namely Karl Popper and Thomas S. Kuhn. Popper's insistence that scientific theories and hypotheses be falsifiable (e.g., Popper 1935) is too strict a requirement even for most nursing theory or science, not to mention nursing itself.^1^ Although hypothesis testing is essential in some parts of nursing science, it does not align with work that falls within the hermeneutical tradition. Admittedly, this observation may be used to criticize nursing science and argue that it should adopt a more Popperian approach. Still, such a conclusion seems premature if we consider that Popper is mainly interested in the natural sciences. The problem, then, is that Popper's account of science is too narrow in scope. Similarly, it is debatable whether nursing science is, or should be, normal science in the sense of Thomas S. Kuhn (1962).^2^ As some scholars have argued, there is no clear‐cut paradigm for nursing science. Instead, nursing science appears to be multi‐paradigmatic (Younas and Parsons 2019) or guided by a meta‐paradigm (Fawcett 1984; see Bender 2018, for discussion). It is again debatable whether this has any consequences for the status of nursing or nursing science. The reason is, again, that Kuhn's theory focuses on the pure natural sciences. As far as his account is based on insights from the history of science, he mainly draws on examples from physics, astronomy, and chemistry. Accordingly, it is dubious whether Kuhn's approach should be applied to nursing science.

In this paper, we propose a fresh start. We examine the extent to which nursing is scientific by applying a new account of science introduced by Hoyningen‐Huene (2013). This account has a broad scope and, therefore, seems better suited for discussing the scientific nature of nursing. The account applies to all disciplines that are taught at major research universities and fall under the German term “Wissenschaft,” which is broader than the English term “science.” In the following, we shall use “science” in this broad sense, including, for instance, mathematics, law, and cultural studies as done by Hoyningen‐Huene (2013, 8).

Why is it important to apply Hoyningen‐Huene's account of science to nursing? Currently, nurses have strong motivations to argue that their profession is scientific. Science not only enjoys high esteem in society quite generally. By emphasizing that their profession is grounded in science, nurses can also defend the high quality of their work. Furthermore, if nursing is based upon science, nurses can justify why they need training at universities. However, nurses' claims to scientific knowledge are not successful if they draw on views of science that are outdated or yield dubitable results when applied to nursing. For this reason, we use a new and more convincing account of science to understand the scientific nature of nursing.

Our analysis can also make a positive difference to the self‐understanding of nurses and aid their reflection on their profession. Furthermore, our results indicate what may be done if nursing is to become even more scientific. Still, the focus of our paper is not on proposals to advance nursing or nursing science, but rather on a thorough analysis of the scientific nature of nursing today. Note also that we focus entirely on those characteristics of nursing that contribute to its being scientific. We are not interested in a comprehensive definition of nursing science (e.g., Grace and Zumstein‐Shaha 2019, 1) because such a definition would also have to specify what the topic of nursing science is and how it differs from other sciences, particularly medicine (see Fedyk 2023 for a recent account).

We proceed as follows: In Section 2, we briefly describe Hoyningen‐Huene's account of science. We apply it to nursing in Section 3. We discuss our findings in Section 4.

## 2 Systematicity: Hoyningen‐Huene's Theory of Scientific Knowledge

In the background of Hoyningen‐Huene's approach is skepticism about the idea that science can be characterized using timeless necessary and sufficient conditions unambiguously defining science. This skepticism was prominently expressed by philosopher Larry Laudan who wrote: “The evident epistemic heterogeneity of the activities and beliefs customarily regarded as scientific should alert us to the probable futility of seeking an epistemic version of a demarcation criterion.” (Laudan 1983, 124; emphasis deleted). Hoyningen‐Huene would agree that science cannot be characterized using an ahistorical definition that captures the “nature” of science (cf. Hoyningen‐Huene 2013, 10 f.); he further denies a sharp boundary between science and nonscience (ibid., 11–13). He still takes it that we can characterize science in some way, as is evident from his central thesis:

Scientific knowledge differs from other kinds of knowledge, in particular from everyday knowledge, primarily by being more systematic.

ibid., 14

Let us explain this claim. First, science and nonscience are distinguished at the level of knowledge. The question that the thesis answers is whether knowledge is scientific or not. Other things, such as hypotheses, theories, methods, and experiments, are commonly qualified as scientific, too (see Godfrey‐Smith 2021, Sect. 4.6 for an illuminating discussion). However, none of these things are central to Hoyningen‐Huene's approach. The focus on knowledge is natural because knowledge is probably the most essential product of science. As we shall see, focusing on knowledge also allows for considering all knowledge‐seeking practices in science. Here, the term ‘knowledge’ refers to so‐called propositional knowledge: knowing that something is the case (ibid., p. 21). Hoyningen‐Huene takes knowledge to be what counts as knowledge (2013, 21). Accordingly, in his approach and our paper, knowledge need not be true or correct. This is an advantage because many things that were considered scientific knowledge turned out to be false. Still, more systematic – and thus possibly scientific – knowledge will often be closer to the truth because it is more systematically justified.

Second, Hoyningen‐Huene compares scientific knowledge to knowledge that is considered *legitimate*. This is a major departure from the traditional focus of philosophy of science. Previously, philosophers attempted to distinguish real science from metaphysics and pseudo‐science, thereby separating it from forms of knowledge they typically deemed problematic or illegitimate. As formulated in the central thesis, one significant contrast to scientific knowledge is everyday knowledge such as knowledge about where my friends live and what they are like. Similarly, professional knowledge that is not yet scientific can also differ from scientific knowledge. For instance, bakers know how the ingredients of bread behave and how they need to be stored. However, for a long time, this knowledge has not been subjected to scientific inquiry. Hoyningen‐Huene's thesis then states that scientific knowledge in this particular domain, if it exists, is more systematic than professional knowledge in this domain (see Scholz 2020, 14–15). This point will be relevant in our discussion of nursing knowledge.

Third, the central thesis is comparative rather than categorical (Hoyningen‐Huene 2013, 22). The idea is not that scientific knowledge has a feature, namely systematicity, that other kinds of knowledge lack entirely. Instead, systematicity is assumed to be a matter of degree, and scientific knowledge differs from other types of knowledge by its comparatively higher degree of systematicity. This suggests that the transition between nonscience to science is smooth rather than sharp.

Fourth, the main thesis is meant to be topic‐ or domain‐specific: Whether some knowledge is scientific depends on how it compares to other knowledge *about the very same topic or within the same domain*. Accordingly, knowledge about the French Revolution can be scientific even if it is not systematic in the sense in which mathematical knowledge is. To qualify as scientific, it must only be more systematic than other, notably everyday knowledge about the French Revolution. The domain‐specificity of Hoyningen‐Huene's claim is healthy because it does not imply that all sciences are subject to the same standard of systematicity.

Fifth, the thesis allows that scientific status depends on time. For instance, according to the thesis, alchemy may have contained scientific knowledge centuries ago because it was more systematic than any other knowledge about its topic at that time. Still, today, alchemy no longer qualifies as scientific because knowledge in current chemistry is much more systematic in comparison.

Finally, and most importantly, according to Hoyningen‐Huene, the key concept used to characterize science is systematicity. This raises the question of what systematicity is. Hoyningen‐Huene answers this question by first contrasting systematicity with opposites such as randomness or disorder (ibid. 26 f.). However, this does not sufficiently explicate systematicity. He thus proposes that systematicity needs to be clarified along several dimensions. The dimensions are key aspects of science or achievements that scientific knowledge can attain (ibid. 27):

a. description
b. explanation
c. prediction
d. defense of knowledge claims
e. critical discourse
f. epistemic connectedness,
g. completeness as ideal
h. knowledge generation
i. representation of knowledge.

To illustrate two dimensions very briefly, we can say that descriptions (a) in biology are more systematic than in daily life because they draw on systematic classifications using taxa such as species or family. Similarly, explanations (b) in physics are more systematic than everyday explanations of physical phenomena because they use physical theories such as Einstein's theories of relativity. What exactly systematicity is varies with the dimensions and the disciplines under scrutiny (ibid. 28 f.). It is not assumed that every dimension applies to every scientific discipline. For instance, prediction is not a task of literary studies. For the latter to be scientific, it is sufficient that it is more systematic than other forms of knowledge in the dimensions that are relevant to it (ibid., 36).

## 3 Applying Hoyningen‐Huene's Theory to Nursing

To apply Hoyningen‐Huene's account to nursing, we have to prepare the ground. First, we have to explain what exactly we will assess for scientificity. Since Hoyningen‐Huene's account focuses on knowledge, we must specify which kind of knowledge we will analyze regarding scientificity. Second, since the approach involves a comparison, we must determine an appropriate contrast class.

The kind of thing we want to assess for scientificity is, of course, nursing. However, we must distinguish between nursing practice and nursing science. It makes a difference whether a person engages in nursing practice, taking care of patients and documenting their work, or whether they conduct nursing science, setting up a randomized controlled trial experiment, developing new theories, or publishing results. Of course, the distinction is not strict: a nurse in a hospital may perform care work that also counts as research because it is documented and assessed as part of a larger research project. Still, a distinction can be drawn using the primary aim of some action: is it the care of specific patients or the acquisition of knowledge and understanding? In the latter case, the primary objective is to discover and consolidate knowledge with a broader scope; thus, persons with this primary goal tend to leave the immediate care of patients to others. When discussing whether medicine is scientific, according to Hoyningen‐Huene, Lyre (2018) draws a similar distinction between medical practice and medical research. He maintains that medical doctors utilize a great deal of scientifically developed knowledge in their daily work. Still, when treating patients, the medical doctors do not conduct research. By contrast, medical scientists specifically develop research questions and respective studies to gain new knowledge in a systematic way (Lyre 2018, 2).

Given the distinction between nursing science and nursing practice, we may wish to investigate to what extent nursing *science* is scientific. Such an investigation is not as trivial as it may sound. Although we speak of “nursing science,” a closer investigation may be required to determine whether it truly is a science. Nevertheless, in this paper, we will focus on nursing *practice*. One reason is that it is more challenging to address the question of whether nursing practice is scientific. It would be strange if nursing science, a discipline now taught at universities, did not instantiate important traits of science. Things are less straightforward when it comes to nursing practice. Additionally, the scientific nature of nursing practice is crucial in justifying the demand that nurses should have a university education. Note, however, that to the extent that current professional nursing practice is informed by current nursing science, our investigation of professional nursing practice will also consider nursing science.

Nurses need several kinds of knowledge for their practice (Carper 1978; Chinn et al. 2021). According to Chinn et al. (2021), there are five types of knowledge: emancipatory, ethical, personal, aesthetic, and empirical. Nursing science may be called upon to ground all these patterns of knowing. However, nursing science does not encompass or support all knowledge of nurses, nor does it include their entire empirical knowledge. For instance, nurses obtain knowledge about individual patients by asking them about their suffering or measuring their blood pressure, or they draw on empirical knowledge from other disciplines, particularly medicine or psychology.

For our present study, we wish to consider the full breadth of knowledge used in professional nursing practice. In particular, we will consider the knowledge nurses gain in their practice. However, as other scholars have pointed out, not all kinds of knowledge that nurses possess are, or can be, scientific. Hoyningen‐Huene only considers propositional knowledge – knowledge that something is the case (2013, 21). Most scientific knowledge falls into this category. However, the restriction to propositional knowledge excludes practical knowledge of how to do something, for instance, how to perform an injection (see Ryle 1946 for this). Other parts of nurses' knowledge may, in principle, be investigated using Hoyningen‐Huene's approach, but still not turn out to be scientific. Often, this is as it should be because we would not call those parts of knowledge scientific pre‐theoretically. For instance, even though personal knowledge is to some extent knowledge that something is the case, say, that I tend to get nervous when children cry, knowledge of this type is not the kind that is developed and published in scientific research. Consequently, our analysis of nurses’ knowledge will not apply to all patterns of knowing in nursing, nor will it distinguish all nursing knowledge as scientific. For instance, aesthetic knowledge, as described by Chinn et al. 2021, 9–10), does not lend itself to a scientific systematization because it is concerned with the “uniqueness of meaning in a [specific] care situation” (ibid. 9). To keep the project of this paper manageable, we will not delineate the patterns of knowing that may be analyzed using Hoyningen‐Huene's theory and which types of nursing knowledge qualify as scientific according to his theory. Our aim is more modest: We will simply give examples of nursing knowledge that is scientific according to Hoyningen‐Huene's approach. Part of this knowledge is certainly empirical. However, we also think that parts of nurses' ethical knowledge can be scientific. In any case, we do not mean to downplay the importance of those parts of nurses' knowledge that are not scientific.

To apply Hoyningen‐Huene's account of science, we must also clearly determine the comparandum to current professional nursing practice and its associated knowledge. For the purposes of this paper, we choose as a contrast the knowledge of nurses before the consolidated views on nursing knowledge as proposed in the Consensus Statement on Emerging Nursing Knowledge in 1998 (Butts and Rich 2011; Roy and Jones 2007). This statement arose from an effort to achieve consensus on the development of nursing knowledge. What matters for our purposes is not the content of the statement (reprinted in Roy 2007) but rather the state of the knowledge a couple of years before 1998. At this time, evidence‐based nursing was still in its infancy. Many classifications were just emerging. Additionally, the implementation of new knowledge into practice was less thoroughgoing than it is today. Similarly, investigations into nursing research were slowly evolving, whereas nursing education was more guided by nursing theory (Gortner 2000; Stolley et al. 2000; Mackey and Bassendowski 2017). But then, “[b]y the early 1990s, the nursing profession moved away from thought traditions that emphasized interpersonal relationships and the therapeutic use of self and began to emphasize nursing outcomes rather than process. […] Nurses no longer expected themselves to be cap‐and‐stockings‐clad assistants, but rather innovators, scientists, and scholars” (McMenamin et al. 2019). We are interested in how nursing knowledge after this development differs from that before it. Knowledge from the time before this development matured also seems to be a good proxy for the knowledge or, instead, the beliefs that lay people currently have about nursing, for instance, the knowledge that laypersons have or acquire when caring for sick relatives without having professional nursing training.

Of course, the 1998 statement does not mark a sharp transition between two kinds of nursing practice. It is merely one step in the continuous development of nursing science, which is often traced back to Florence Nightingale, among others. Since then, nursing has increasingly become informed by research. There has been a shift from apprenticeship to university‐based education in the nursing profession, and a nursing science discourse has been gradually established. The developments have, of course, differed between countries. Still, to a good approximation, we can say that nursing practice today is different from that before 1998. This is because, until the late 1990s, nursing science was less developed and was only sporadically applied in practice (Butts and Rich 2011; Roy and Jones 2007). Following the quest for evidence‐based medicine, in the 1990s, evidence‐based nursing gained traction (e.g., Wallace et al. 1997; Ervin 2002) and was put into practice, as is evident from related guides (e.g., Ackley and Ladwig 2007) and guidelines (e.g., DiCenso et al. 2005; see Beyea and Slattery 2013 for a historical account).

Ultimately, our results do not significantly depend on the precise choice of the baseline for the comparison, viz., the Consensus Statement. As mentioned earlier, there is a significant trend toward basing nursing on evidence and demonstrating nursing's key contributions to patient outcomes. Accordingly, it does not matter where exactly the baseline for the comparison is set, as long as it is significantly before the present time, such that there are significant differences in systematicity.

In sum, in what follows, we will investigate to what extent and in which sense the knowledge used in today's professional nursing practice is more systematic and thus scientific than nursing knowledge before the Consensus Statement. With this in mind, we can now go through the dimensions. To simplify our presentation, we will not always explicitly contrast nursing knowledge today with that before the Consensus Statement, but instead characterize current nursing knowledge using its state and recent developments.

### 3.1 Description

Description is a key part of professional nurses' work. Patient history and assessment constitute the description of patients' conditions. In documenting their work, professional nurses describe the interventions and the outcomes following the nursing process. Descriptions of these sorts are needed specifically in collaborative work. They also facilitate the monitoring and assessment of caring processes.

According to Hoyningen‐Huene (2013, Sect. 3.1), there are various ways in which descriptions can become more systematic. A crucial means to achieve systematicity is to utilize systematic classifications. Such classifications have become frequent in professional nursing. For instance, many patient outcomes can be classified using the Nursing Outcome Classification (NOC; Johnson and Maas 1998; Moorhead et al. 2023). The NOC taxonomy comprises seven domains, including functional health and psychosocial health, each of which is divided into classes such as mobility and self‐care for functional health (Moorhead et al. 2023, pp. 58‐9). The classes contain lists of outcomes, such as 0213‐Joint Movement: Ankle and 0213‐Joint Movement: Elbow, among others (ibid., 61). Likewise, nursing interventions are classified in the Nursing Intervention Classification (NIC; Dochterman et al. 2018). Both the NOC and the NIC have been developed based on scientific evidence. In recent years, the ICNP (International Classification of Nursing Practice) has gained even greater importance. The classifications in this system inform SNOMED CT, an electronic taxonomy that informs many electronic documentations (International Council of Nurses ICN 2019).

According to Hoyningen‐Huene (2013, Sect. 3.1.5), descriptions can also be rendered more systematic through the use of quantitative statements. Quantitative descriptions are indeed common in contemporary nursing. Nurses describe their patients' conditions using measurements of body temperature, for instance. For a more advanced example, consider ineffective health self‐management. In a meta‐study, da Silva et al. (2022b) consider clinical indicators for ineffective health self‐management and stress that one of them, failure to take action, can be measured using the degree of physical activity and other characteristics.^3^ Similarly, the NOC proposes measuring various outcomes to determine ineffective health self‐management in individuals with diabetes. Among these, a Likert scale can be used to determine the extent to which a person with diabetes adheres to the recommended regimen (Oh and Moorhead 2019; Lee et al. 2019).

When discussing the systematicity of descriptions, Hoyningen‐Huene (2013, Sect. 3.1.6) also mentions empirical generalization. There is a great interest in generalization in nursing science. For instance, it would be helpful to know how nurses' working conditions correlate with patient outcomes in general (see Stalpers et al. 2015 for a related meta‐study). As a concrete example, we mention the STRAIN study (Peter et al. 2020a, 2020b), which offers quantitative generalizations on the relationship between working conditions, on the one hand, and job satisfaction and health‐related outcomes, on the other, for nurses and other healthcare professions in Switzerland (Peter et al. 2020a, 2020b). They found that “[h]ealth professionals without management responsibilities reported the poorest working conditions in relation to various stressors, job satisfaction […] and health‐related outcomes” (Peter et al. 2020b, 969). This result is quite general because it does not refer to a single hospital, but rather concerns the entire country of Switzerland.

Overall, it is fair to say that present‐day professional nursing uses classifications, quantification, and generalization more often than was common until the late 1990s. In this sense, the descriptions, or descriptive knowledge, used and produced in nursing are more systematic than they used to be in the 1980s.

### 3.2 Explanation

Nurses often have to explain to patients what they are doing and what specific terms mean, but this is not what Hoyningen‐Huene means when addressing explanation as a dimension in which systematicity may unfold (2013, Sect. 3.2). Rather, he refers to the explanation of phenomena. Such explanations answer why‐ or how‐questions, for instance, “Why did a pressure ulcer arise?” Explanations typically go beyond mere descriptions of a phenomenon to offer insight into its origin or underpinnings.

A very basic example of explanation in nursing is the nursing diagnosis, which forms the basis of any successful nursing treatment. Nursing diagnoses have the potential to explain a range of symptoms in terms of one underlying cause. In current professional nursing, diagnoses are particularly systematic because they are based upon systematic taxonomies. At this point, the NANDA‐I classification or similar ones are most relevant (Herdman et al. 2024; see Strudwick and Hardiker 2016; Othman et al. 2020, and Rodrigues et al. 2022 on the use of classifications in nursing). The NANDA‐I taxonomy is particularly interesting because it is linked with the NIC and the NOC. To elaborate on one example, let us consider the nursing diagnosis “ineffective health self‐management”. It is defined as “unsatisfactory management of symptoms, treatment regimen, physical, psychosocial, and spiritual consequences, and lifestyle changes inherent in living with a chronic condition” (Herdman et al. 2024, 214). The entry for this diagnosis describes the associated characteristics, possible consequences, and common factors that accompany the problem. In addition, the populations with a risk of developing this condition are listed together with associated health conditions (Herdman et al. 2024, 215). The information provided by nursing diagnoses is reliable as they are based on scientific evidence, nursing theory, or other theoretical resources from related scientific disciplines (Herdman et al. 2024). Such a taxonomy enables nurses to identify diagnoses and, consequently, provide explanations systematically.

Explanations can also be given in terms of theories. Such explanations are particularly systematic because typical scientific theories are highly systematic, and theories with a broad scope facilitate explaining a wide range of phenomena using the same explanatory framework (see Kitcher 1981 for this idea). In nursing science, several theories have been developed with various scopes. Examples include the Self‐Care Deficit Theory of Nursing by Orem (1971, with revised editions of this book), the Humanbecoming Theory by Parse (1981, 1992, 1999), and the Omnipresence of Cancer Theory by Cox and Zumstein‐Shaha (2018). It is generally acknowledged in nursing that theories in this domain incorporate and condense empirical evidence, thereby providing the necessary evidence for practice (Fawcett and Garity 2009; Fawcett 2017). Note, though, that not all of these theories offer explanations as such; the primary aim of some such theories is rather often to deliver a framework for constructing explanations, as is the case with the Omnipresence of Cancer Theory by Zumstein‐Shaha et al. (2018; see also Zumstein‐Shaha, Cox and Fawcett 2020). This theory describes the experience of being diagnosed with cancer. Based on this description, key factors are provided that subsequently allow for explanations of specific patient behavior following the disclosure of a cancer diagnosis (see Cox and Zumstein‐Shaha 2018, for more details). A more thorough investigation of nursing theories is beyond the scope of this paper.

Another form of more systematic explanation is reductive explanation (Hoyningen‐Huene 2013, 68). The latter explains phenomena described at one particular level by using a different level of description. For instance, a mechanism underlying the phenomenon of wound healing is provided to explain this very phenomenon. This is uncommon in nursing, where much practice does not move to the biochemical level, for instance. Still, some attempts in this direction can be identified in nursing. For example, the allostatic load can be used to explain “stress‐induced biological risk” (see Szanton et al. 2005; Guidi et al. 2021). Arguably, reductive explanations are not very prominent in nursing because they are in tension with the holistic approach that is popular in nursing.

In summary, mainly due to recent classifications, explanations in current nursing practice are more systematic than those used previously. Note that this does not mean that the use of classifications is always beneficial. Our argument is only that a particular kind of knowledge, namely diagnostic knowledge, is more systematic and thus scientific if it is based upon classifications.

### 3.3 Prediction

Predicting patient outcomes and treatment side effects would be of high interest to nursing practice. However, prediction in nursing is notoriously difficult because many factors typically influence outcomes. Human health and related care measures are not subject to the same kind of simple laws as they are known in some branches of physics. Accordingly, nursing does not commonly provide a patient with a detailed prognosis. Still, nurses try to anticipate outcomes and side effects of treatments, at least as likely events or possibilities. Anticipations of this kind are particularly systematic in current professional nursing, where they can be built upon classifications.

Consider again the nursing diagnosis of “ineffective health self‐management.” The entry in the NANDA‐I classification lists populations at risk and associated conditions. If nurses encounter a situation in which they observe several characteristics that point to this particular nursing diagnosis, they can expect some related conditions. In our concrete example, health outcomes such as “having difficulties keeping the prescribed regimen” or “failure to take action to reduce risks” can be expected (da Silva et al. 2022b, p. 494).

As Hoyningen‐Huene (2013) elaborates, systematic predictions can be obtained using theories and models. Not all nursing models or theories allow for predictions. For instance, the Uncertainty in Illness theory (Mishel 1988) is essentially a causal model that posits specific qualitative causal relationships. Since the strengths of the causal connections are not quantified, the theory cannot be used to make precise predictions on the values of variables such as adaptation. Despite this, models and theories based on empirical research, such as the Roy Adaptation Model (Roy et al. 2009), or theories drawing heavily on other disciplines, like the Theories of Uncertainties in Illness (Clayton and Dean Kruzel 2024), facilitate predictions (Jairath et al. 2018).

Therefore, to the extent to which predictions are cast in current professional nursing, they are more systematic than in previous nursing practice because sources such as the NANDA‐I taxonomy or specific nursing theories are employed.

### 3.4 Defense of Knowledge Claims

According to Hoyningen‐Huene, scientific knowledge is more systematic than other knowledge in that it is more systematically defended. For instance, in the empirical sciences, hypotheses are systematically examined using controlled experiments (Hoyningen‐Huene 2013, 98–102), and scientists try to replicate the results of experiments.

With the promotion of evidence‐based nursing, the defense of knowledge claims has become more important in nursing. Evidence‐based nursing means that nursing practice is based on the latest evidence. In Switzerland, for example, it is by law required that interventions be efficient, adequate, and economical. If it can be demonstrated that these conditions are not met, insurance companies may refuse to cover the costs (Federal Assembly of Switzerland 2024).

We can provide a concrete example by referring again to the previously selected NANDA‐I diagnosis of “ineffective health self‐management”. This particular diagnosis emerged from a systematic review of existing evidence, that is empirical studies, regarding patients who do not adhere to the recommended treatment or a similar regimen (da Silva et al. 2022a). Drawing on the evidence, it was possible to define the diagnosis, to elicit its characteristics, related factors, populations at risk, and associated conditions (da Silva et al. 2022a). In addition, this diagnosis was validated using scientific methods (Oh and Moorhead 2019). The validation demonstrated that this nursing diagnosis can be used to effectively identify patients with ineffective health self‐management (Oh and Moorhead 2019, p. 228).

In current nursing science, evidence is gathered systematically because empirical data are collected and analyzed using validated and reliable methods that are also used in other sciences. These methods prohibit the use of merely anecdotal evidence and require a thorough investigation of possible errors. All this makes the defense of knowledge claims more systematic. Accordingly, to the extent that nursing practice draws upon results from nursing science, it is built upon knowledge that is more systematic than everyday knowledge. It must be acknowledged, however, that there are still areas of nursing practice that are not fully grounded in the most recent evidence (see below).

Nevertheless, overall, there is no doubt that the knowledge used in current nursing practice is more systematically defended than before the late 1990s. The standards for evidence needed to support nursing practice have become widely accepted. But what about the knowledge that is *gained* within current nursing practice? It seems fair to say that knowledge obtained in current nursing practice is also more systematically defended than in the 1990s or elsewhere. For instance, key characteristics such as temperature and blood pressure are measured in a much more regular and reliable way due to protocols and professional measurement devices. Still, in nursing practice, there are limits to the defense of knowledge claims. If a specific diagnosis has gained sufficient credibility, it is time to act rather than further investigate the patient's condition.

### 3.5 Critical Discourse

According to Hoyningen‐Huene, the increased systematicity of scientific disciplines not only refers to knowledge itself but also extends to the social life of science, that is, its formal and informal customs of knowledge‐seeking, or its social institutions, as sociologists would put it. The main point is that the sciences have developed numerous norms and institutions that foster critical discourse: everything proposed in science will undergo critical scrutiny within the pertinent scientific community. This is not to deny that there is critical discourse outside science. However, in everyday life, critical discussion often occurs unintentionally and not in a planned or institutionalized manner. Thus, critical discourse in the sciences is much more systematic than in everyday discourse. Note that this dimension of systematicity is connected to the previous one: The dimension “critical discourse” translates the *epistemic* dimension “defense of knowledge claims” into the *social* institutions of science.

There is no doubt that nursing science is highly systematic regarding critical discourse. Nursing science is organized and practiced in a similar way to other sciences. For instance, publications in high‐ranking journals require peer review. Notably, the first scholarly nursing journal, Nursing Research, was first published in 1952 (Donahue 2011). There are many conferences where contributions must undergo a peer‐review process and/or face critical questions. Accordingly, nursing practice based on nursing science utilizes knowledge that is more systematic in the dimension of critical discourse than other forms of knowledge.

But what about knowledge that is obtained in nursing practice? Are there rules or incentives that foster critical examination of the knowledge that nurses think they have received in practice? This is less clear. True, some nurses attend journal clubs (see Steenbeek et al. 2009; Ritter‐Herschbach et al. 2022; Duffy et al. 2023), and there are several symposia, conferences, and other direct meeting opportunities available for every level of nursing and specialty. In addition, various associations around the globe such as the International Council of Nurses, the American Academy of Nursing, and the Swiss Nurses' Association unite nurses to address nursing practice, science, and other relevant areas including genomics, informatics, and policies. Still, such institutions often primarily foster the transmission of knowledge rather than engaging in critical debates on the findings of nurses. However, case presentations may be more akin to a critical discussion, particularly when conducted in an interprofessional setting. Similarly, policy dialogues as held by the American Academy of Nursing, seem to foster critical discourse more systematically (American Academy of Nursing 2023).

In summary, there is no doubt that nursing science engages in critical discourse, similar to many other sciences. It is less clear that the institutionalization of nursing incentivizes critical scrutiny of knowledge claims to the same degree (cf. Andrade et al. 2019).

### 3.6 Epistemic Connectedness

Things are pretty similar regarding the next dimension, epistemic connectedness. Hoyningen‐Huene has introduced this dimension to explain why specific research conducted for courts of law, in business companies, or journalism does not count as scientific. The reason is that this research is not systematically connected to scientific research: For instance, journalists working on a recent government crisis or the political reactions to a drought typically do not investigate how their findings may be embedded into political theories and relate to results from other scientific fields. This is evident from the fact that their articles do not refer much to scientific work. The reason is often that their readers are more interested in specific cases rather than general theories. In contrast, scientific research is more systematically embedded in other relevant scientific studies and aims to contribute to the integration of its findings into a comprehensive body of knowledge. Epistemic connectedness is often a matter of generality, especially in disciplines that aim at general models, laws, and theories (Hoyningen‐Huene 2013, 113–124).

Research questions in nursing science possess some generality, enabling them to be systematically connected to other research within nursing science, as well as to other fields such as medicine and social sciences. Thus, when nursing practice draws on knowledge from nursing science, it utilizes knowledge that is systematically connected to other scientific knowledge. It is a bit different with knowledge gained by practicing nurses. This knowledge is often very case‐specific, and nurses do not have the time to connect their findings to existing scientific research. Their focus is typically on the care of the individual patient, rather than contributing to the development of a systematic science. Admittedly, in interprofessional teams, nurses connect the knowledge they gain to other findings. For instance, they try to connect it to medical diagnoses; conversely, they communicate their finding that a specific patient has expressed pain when being turned to medical doctors or social workers. Still, connections of this kind do not significantly contribute to epistemic connectedness, as defined by Hoyningen‐Huene, because they do not establish connections to *scientific* knowledge.

### 3.7 Completeness of Knowledge

Another dimension in which systematicity plays out is the completeness of knowledge. According to Hoyningen‐Huene, scientific knowledge is more systematic than everyday knowledge because it follows the ideal of completeness more strongly (2013, 3.7). One of his examples refers to classifications: they are supposed to be exhaustive or to cover everything from a specific domain of things (2013, 129).

The ideal of complete coverage is certainly operative in nursing. For example, the NANDA diagnoses started with a large selection of diagnoses in the early 80ies. Since then, NANDA diagnoses have been constantly updated and expanded. Chapter 1 of the latest NANDA‐I diagnoses publication provides a comprehensive overview of newly integrated diagnoses, updates, and retired diagnoses (Herdman et al. 2024). When considering our previous example of a diagnosis, that is ineffective health self‐management, it is stated that this diagnosis was only introduced and approved in 2020 (Herdman et al. 2024). Overall, evidence for all nursing phenomena encountered in practice is expected to be further developed in the future (Beyea and Slattery 2013; Mackey and Bassendowski 2017).

Knowledge‐gaining practices within nursing are less obviously driven to completeness. Still, upon closer examination, we can observe that a quest for completeness is at work when nurses are asked to think more holistically and conceive the person as multifaceted, that is physical, psychological, social, cultural, and spiritual (Fawcett 2017; Roy et al. 2009).

We can thus conclude that the knowledge used in nursing is subject to the ideal of completeness, as far as this is possible, and that nurses strive towards completeness when seeking a holistic appreciation of their patients' states. We can, furthermore, infer that current nursing is more systematic than our baseline if the quest for completeness has become stronger in the decades after the Consensus Statement. Admittedly, it isn't easy to demonstrate that this condition is fulfilled, as it essentially involves the strength of an ideal. Still, the fact that a book titled “Holistic nursing: A handbook for practice” (Dossey et al. 1988) appeared no earlier than 1988 and has since then seen several new editions – the currently latest being the eighth edition of 2020 – may be considered as evidence that the quest for a holistic view has become stronger.

### 3.8 Production of New Knowledge

Hoyningen‐Huene's next dimension refers to how science strives to produce new knowledge: Science is more systematic in its efforts to obtain new knowledge than is the case in everyday life (2013, 3.8). In this regard, Hoyningen‐Huene emphasizes the generation of data and attempts to apply knowledge from other disciplines.

In both respects, nursing science parallels other sciences in being more systematic than everyday inquiry. As Westra et al. (2017) show, scholarly nursing research is now sometimes based on big data, which involves the systematic production of a large amount of knowledge. Earlier, Brennan and Bakken 2015, 477) have argued that “Big data and data science have the potential to provide greater richness in understanding patient phenomena and in tailoring interventional strategies that are personalized to the patient.”

In its quest for more knowledge about nursing phenomena, nursing science also heavily draws on methods from other disciplines. For example, to determine the levels of quality of life, valid and reliable questionnaires such as the Euroqol Five‐level Questionnaire are used (Okajima et al. 2013). The EuroQol 5‐level questionnaire is an instrument that has been tested through interdisciplinary scientific research (e.g., Herdman et al. 2011). Similarly, validated and reliable instruments such as the Distress Thermometer are used in oncology practice to determine stress levels and burden levels in cancer patients. By using these methods, nursing science significantly extends its database. The resulting data can then be used in nursing research to inform nursing practice (Götz et al. 2020, 2024; Kirk et al. 2021).

In nursing practice, the production of knowledge is also quite systematic. The nursing process is crucial at this point. Consider, for instance, how the American Nurses Association describes assessment, the first step of the process:

An RN uses a systematic, dynamic way to collect and analyze data about a client, the first step in delivering nursing care. Assessment includes not only physiological data, but also psychological, sociocultural, spiritual, economic, and lifestyle factors as well. For example, a nurse's assessment of a hospitalized patient in pain includes not only the physical causes and manifestations of pain, but the patient's response—an inability to get out of bed, refusal to eat, withdrawal from family members, anger directed at hospital staff, fear, or request for more pain mediation [sic].

American Nurses Association n.d.

This description not only contains the term “systematic”. It also emphasizes that the assessment encompasses various aspects of the patient and their situation. Accordingly, in its first step, the nursing process encourages nurses to increase their knowledge systematically.

In this context, nursing theories can prove helpful. Many of them are or provide a systematic framework to consider nursing phenomena from various angles. On this basis, data gathering in the nursing process is carried out even more systematically and the subsequent choices of treatments can be based on evidence (Fawcett and Garity 2009; Fawcett 2017).

We thus conclude that the process of knowledge generation used since the Consensus Statement in 1998 is very systematic, both in nursing science and in nursing practice. Since instruments such as the Euroqol Five‐level Questionnaire are constantly elaborated, it seems fair to say that knowledge generation is also more systematic than it was before the Consensus Statement.

### 3.9 Representation of Knowledge

Scientific knowledge also excels over other kinds of knowledge in terms of systematic presentation. This is Hoyningen‐Huene's central thesis concerning the last dimension. He mentions scientific nomenclatures, formal languages in logic, a depiction of the tree of life, and maps from geography as examples (Hoyningen‐Huene 2013, 142–149).

It is easy to find systematic representations of knowledge in nursing science. For instance, many nursing theories are represented with viewgraphs. For a concrete example, see Figure 1 in Zumstein‐Shaha et al. (2020, E123). This viewgraph illustrates the primary concepts from a theory and their interrelationships. However, viewgraphs or infographics have been used in nursing long before the Consensus Statement. A famous example is Florence Nightingale's “Diagram of the causes of mortality in the army in the East” shown in Martineau and Harriet (1859).

For evidence that the representation of knowledge has become more systematic in recent years, we consider the ISBAR scheme. The latter is a situational briefing tool designed to facilitate communication among healthcare professionals (Marshall et al. 2009; see also Burgess et al. 2020). The professionals are requested to describe their identity, the situation, the background, and their assessment of the situation and to provide a request. ISBAR thus encourages practitioners to present information in a clear, organized, and systematic manner. Empirical evidence has shown that using ISBAR leads to more comprehensive and clearer presentations (ibid.). Interestingly, ISBAR is designed for practice and is concerned with the representation of local, case‐specific knowledge rather than scientific knowledge.

As a second example, we mention standardized nursing terminologies. They go hand in hand with classifications, which we have already discussed. This is evident from the name of the “Task Force to Name and Classify Nursing Diagnosis,” which first met in 1973 (Jones et al. 2010). Hoyningen‐Huene (2013, 142–143) likewise notes the interconnectedness between classification and attempts to provide a systematic terminology using a nomenclature. While classification focuses on organizing items, such as diseases or treatments, into classes and subclasses, nomenclature is concerned with a system of terms that reflects the taxa. Over the last few decades, standardized nursing languages have been developed in parallel with classifications such as the International Classification of Nursing Practice, the Omaha System, and the Clinical Care Classification (Jones et al. 2010). Recently, Dos Santos et al. (2024) have called for more action in this direction.

We thus conclude that, after the Consensus Statement, there has been progress in the representation of nursing knowledge. To the extent to which more systematic communication schemes and languages are used, knowledge gained in nursing practice is more systematic than our baseline, which shows the scientific nature of current nursing in the last dimension.

## 4 Discussion

To summarize, we have found that, in Hoyningen‐Huene's dimensions, knowledge in current nursing practice is systematic and often more systematic than in the chosen baseline. This is often the case because knowledge from nursing science is applied, as nurses do when they draw on classifications based on scientific evidence. Knowledge‐gaining practices in current nursing are also more systematic than outside of it. This demonstrates that the knowledge in contemporary nursing is more systematic than other knowledge about nursing and, therefore, more scientific. Accordingly, our recourse to Hoyningen‐Huene's account of science has confirmed the scientific basis of nursing.

Looking back at our argument, we note that classifications such as the NANDA‐I classification have played a significant role. By contrast, theories and models have been found to contribute less to nursing's systematicity. This is interesting, as the 1998 Consensus Statement put a strong emphasis on nursing theories. The significance of theories certainly needs further investigation.

The use of classifications is sometimes met with criticism. One fear is that the peculiarities of individual persons are unduly neglected when classifications are used. For instance, patients differ in their personal projects, and the treatment of the patients should take this into account. Further, Powers (2002) complains that the use of classifications leads to the suppression of valuable evidence and a dominance of scientific, empirical knowledge. It may thus be suggested that our emphasis on classifications has problematic consequences for nursing.

To counter this objection, we would like to clarify the purpose of our argument. We have only argued that nursing knowledge is more systematic if it is based on classifications. We have particularly stressed the importance of classifications in relation to descriptive and explanatory knowledge. Accordingly, we have only demonstrated that these types of knowledge are more systematic and, thus, more scientific when based on classifications. It is a different question whether the classifications are suitable for nursing overall. We do think that the classifications come with advantages. For instance, they facilitate the application of scientific evidence about treatment options and thus improve patient outcomes. They also make communication between nurses easier. In our view, some objections against classifications are based upon misunderstandings. Classifying a disease is not meant to provide a complete description of the patient's condition. Other aspects of a patient's condition have to be taken into account, too, when a treatment is chosen. Further, the NIC is only meant to give conditional advice: If you want to avoid this and this outcome, this and this treatment has a high chance of being effective. This does not mean that this treatment ought to be chosen, all things considered. For instance, the patient may suffer from other problems, or a certain treatment may be incompatible with their personal projects. Personal and ethical knowledge is needed, too, to make such a recommendation. So, our argument is not meant to deny the importance of other kinds of nursing knowledge. Admittedly, in practice, the use of classifications may lead to increased attention to empirical knowledge and, thus, to a neglect of other types of knowledge. However, our argument does not imply that this is a good thing.

To finish our paper, we offer two discussion points.

First, according to our findings, nursing practice is particularly systematic when it is based on nursing science or related scientific disciplines such as medicine or the social sciences. However, it is an empirical rather than a philosophical question how responsive nursing practice is to results from nursing science or other scientific disciplines. Unfortunately, some barriers impede the spread of this knowledge into nursing practice. For instance, there are language barriers. Not all scientific research results are published or available in a language understandable to practitioners, thus translation is necessary. In addition, current nursing care is very much constrained by personnel shortages (Drennan and Ross 2019) and related lack of time for care and for consulting evidence‐based resources to improve care (e.g., Hannes et al. 2007). Note, too, that a significant part of nursing knowledge, particularly personal knowledge, does not lend itself to scientific treatment. Personal knowledge refers to the individual person, while scientific knowledge typically has a broader scope.

Second, although Hoyningen‐Huene's main thesis is meant to be descriptive, it can be used to provide future directions for nursing practice. If the latter is to consolidate its scientific status, systematicity needs to be increased. The results of our paper suggest that the systematicity of nursing is promoted if more knowledge from nursing science is used. Furthermore, systematicity may be fostered by strengthening the role of critical discourse and other habits that promote more systematic knowledge acquisition within nursing practice. Nursing theories are not a must‐have for systematicity, but they promote systematicity when improving prediction and explanation and when guiding data acquisition in nursing practice.

## Funding

The authors received no specific funding for this work.

## Ethics Statement

The authors have nothing to report.

## Conflicts of Interest

The authors declare no conflicts of interest.

## Data Availability

The authors have nothing to report.

## Endnotes

1 For the application of Popper's approach to nursing, see e.g., Allmark (2003), Bergdahl and Berterö (2015), and Guimarães et al. (2018).

2 Applications of Kuhnian ideas to nursing include Adams (1991), Bahramnezhad et al. (2015), Bender (2018), Groer and Clochesy (2020), Guimarães et al. (2020), and Pickler and Dorsey (2022).

3 At this point, this example is relevant regarding the description of failure to take action, but since the latter is a clinical indicator, the example is also relevant to diagnosis, to which we turn below.
